# Early Postoperative Outcomes in Patients with Peripheral Artery Disease Residing in Uranium Legacy-Affected Areas: A Comparative Study

**DOI:** 10.3390/jcm15134994

**Published:** 2026-06-26

**Authors:** Kuralay Ilbekova, Yerbol Dogalbayev, Tairkhan Dautov, Viktor Zemlyanskiy, Tokan Sultanaliyev, Irlan Sagandykov, Alexandr Fursov, Danara Ibrayeva, Farida Bekenova

**Affiliations:** 1 Scientific Research Institute of Radiobiology and Radiation Protection, NJSC “Astana Medical University”, Astana 010000, Kazakhstan; 2 Department of Interventional Radiology, Corporate Fund (CF) University Medical Center, Astana 010000, Kazakhstan; 3 Clinical Academic Department of Radiology and Nuclear Medicine, Corporate Fund (CF) University Medical Center, Astana 010000, Kazakhstan; 4 Center for Vascular Surgery, National Research Oncology Center, Astana 010000, Kazakhstan; 5 Professor G.V. Tsoi Scientific and Educational Center of Surgery, NJSC “Astana Medical University”, Astana 010000, Kazakhstan; 6 Department of Internal Medicine No. 1, NJSC “Astana Medical University”, Astana 010000, Kazakhstan

**Keywords:** lower extremity artery disease, chronic lower limb ischemia, uranium tailings, environmental exposure, Fontaine classification, postoperative outcomes

## Abstract

**Background:** The impact of long-term residence in uranium legacy-affected areas on surgical outcomes in patients with lower extremity artery disease (LEAD) remains insufficiently studied. **Objectives:** This study aimed to compare early postoperative dynamics of chronic lower limb ischemia (CLLI) in patients with LEAD residing in uranium legacy-affected areas and those living outside the zone of potential technogenic exposure. **Methods:** The study included 70 patients with LEAD (CLLI stages IIB–IV according to the Fontaine classification). The study group (*n* = 35) consisted of patients who had resided for at least 5 years within a radius of less than 20 km from uranium tailings storage facilities (Stepnogorsk area, Akmola region, Kazakhstan). The control group (*n* = 35) comprised patients with the same diagnosis living outside this zone. **Results:** The distribution of Fontaine stages was compared between groups before surgery and at 1 month postoperatively. Statistical analysis was performed using Pearson’s chi-squared test. Before surgery, no statistically significant intergroup differences were found in the distribution of CLLI stages (χ^2^ = 3.688, df = 2, and *p* = 0.158). At 1 month after surgery, the control group demonstrated significantly better regression of ischemia stages: mild stages (I–IIa) were observed in 51.4% of control patients versus 8.6% in the study group, while severe stages (III–IV) persisted in 62.8% of the study group versus 22.9% of the control group (χ^2^ = 17.547, df = 4, and *p* = 0.002). Complete regression to stage I occurred only in the control group (2 patients, 5.7%). **Conclusions:** Patients with LEAD residing long-term in uranium legacy-affected areas showed less favorable early postoperative dynamics compared to patients living outside the zone of potential technogenic exposure. The observed association requires cautious interpretation, and further prospective studies with individual exposure assessment are warranted.

## 1. Introduction

Lower extremity artery disease (LEAD) remains one of the pressing issues in contemporary vascular surgery, accounting for a significant proportion of cardiovascular diseases (CVD). Chronic lower limb ischemia (CLLI) resulting from atherosclerotic lesions of the lower extremity arteries leads to progressive deterioration of patients’ quality of life, development of critical limb ischemia, and, in advanced cases, limb loss and disability [1]. Despite considerable advances in revascularization techniques, including endovascular, open surgical, and hybrid approaches, postoperative outcomes remain variable and depend not only on surgical technique but also on age, comorbidities, lifestyle, severity of the atherosclerotic process, and environmental factors [2,3,4].

Residence in environmentally unfavorable regions, particularly near industrial facilities and tailings storage sites, may be considered a factor potentially affecting cardiovascular health [5]. Uranium tailings storage facilities are a source of complex technogenic exposure, including natural radionuclides, external gamma radiation, radon and its decay products, uranium-containing dust particles, as well as associated chemical pollutants such as heavy metals. Such combined exposure may promote oxidative stress, endothelial dysfunction, chronic vascular wall inflammation, microcirculatory disturbances, and reduced tissue reparative capacity, all of which are relevant both for the progression of atherosclerosis and for recovery after vascular interventions [6,7,8,9].

Numerous epidemiological studies indicate an association between long-term exposure to technogenic pollutants, including heavy metals, and an increased risk of coronary artery disease, arterial hypertension, and atherothrombotic complications [10]. However, the impact of residence in uranium legacy-affected areas on the immediate outcomes of surgical treatment in patients with LEAD remains insufficiently studied.

The Republic of Kazakhstan, being a major mining region, hosts a considerable number of waste storage facilities from the mining and uranium processing industries, some of which were established during the Soviet period. In the Akmola region, one of the environmentally significant sites is the tailings storage facility of the Stepnogorsk Hydrometallurgical Plant, formed as a result of uranium ore processing. For decades, mining and processing activities have been conducted in the area of Stepnogorsk and adjacent settlements—Aksu, Zavodskoy, Kvartsitka, and Bogenbay—accompanied by waste accumulation in dedicated storage facilities [11]. A substantial proportion of the population in these settlements resides near these sites, including within a radius of less than 20 km, which allows this territory to be considered a zone affected by uranium legacy sites [12]. Previous radiation–hygiene studies in the Stepnogorsk region have demonstrated the presence of radiation and technogenic exposure factors, including gamma radiation, radon, and uranium contamination of environmental components [11,12,13]. In prior radiation-epidemiological studies, Stepnogorsk, Aksu, Zavodskoy, and Kvartsitka were considered exposed territories, while Akkol, located at a considerable distance from the tailings facility, served as a comparison territory [11,12].

Previous studies conducted in uranium legacy-affected regions have mainly focused on environmental contamination, radiation–hygiene assessment, general health outcomes, cancer incidence, and clinical–epidemiological characteristics of peripheral artery disease. For example, a study from Kyrgyzstan analyzed clinical and epidemiological features of obliterating atherosclerosis of lower limb arteries in patients living near uranium legacy sites and reported more frequent involvement of distal arterial segments and differences in reconstructive procedures among exposed patients [14]. However, early postoperative outcomes after lower extremity revascularization in patients residing in uranium legacy-affected areas remain insufficiently investigated.

Given the above, it is of considerable interest to compare the distribution of chronic lower limb ischemia stages according to the Fontaine classification before and after surgical intervention in patients residing long-term near the tailings facility and patients from environmentally favorable areas. Identifying differences in postoperative dynamics may provide a basis for optimizing management strategies for this vulnerable patient population, including more intensive monitoring and enhanced pharmacological support.

The aim of this study was to compare the early postoperative dynamics of CLLI in patients with LEAD residing long-term in uranium legacy-affected areas and those living outside the zone of potential technogenic exposure.

## 2. Materials and Methods

### 2.1. Study Population

The study included 70 patients with LEAD selected from the overall cohort of individuals who received inpatient treatment in the Department of Interventional Radiology of the Corporate Fund “University Medical Center” (UMC CF) between 2021 and 2024. The total number of patients with LEAD treated in the department during this period was 534.

Inclusion criteria: presence of LEAD; clinical presentation of CLLI stage IIB–IV according to the Fontaine classification [15].

Exclusion criteria: concomitant acute coronary syndrome; acute cerebrovascular accident.

From the overall cohort (*n* = 534), two groups of 35 patients each were formed in accordance with the inclusion and exclusion criteria. The main group (study group, *n* = 35) consisted of patients who had resided for at least the last 5 years in settlements located within a radius of less than 20 km from the tailings storage facility: Republic of Kazakhstan, Akmola region, Stepnogorsk city, Aksu, Zavodskoy, Kvartsitka, and Bogenbay settlements. The proportion of such patients among all those treated in the department during 2021–2024 was 6.6% (35 out of 534).

The control group (*n* = 35) was selected from patients treated in the same department during the same period who had the same diagnosis of lower extremity artery disease and underwent lower extremity revascularization but resided outside the predefined uranium legacy-affected area. The same inclusion and exclusion criteria were applied to both groups. Control patients were included if complete preoperative and one-month postoperative data on chronic lower limb ischemia stage according to the Fontaine classification were available.

Baseline demographic and clinical characteristics of the study and control groups are summarized below. In the study group (*n* = 35), the mean age was 69 years; there were 20 men and 15 women. Diabetes mellitus was present in 19 patients, arterial hypertension in 33 patients, and coronary artery disease in 25 patients. All 35 patients received dual antiplatelet therapy after revascularization. In the control group (*n* = 35), the mean age was 67 years; there were 22 men and 13 women. Diabetes mellitus was present in 15 patients, arterial hypertension in 35 patients, and coronary artery disease in 29 patients. All 35 patients received dual antiplatelet therapy postoperatively. No significant differences in age, sex distribution, prevalence of diabetes mellitus, arterial hypertension, coronary artery disease, or postoperative antiplatelet regimen were observed between the groups (*p* > 0.05 for all comparisons).

#### Characterization of Environmental Exposure

In this study, residence within a radius of less than 20 km from uranium tailings storage facilities was considered a geographic marker of long-term residence in the zone affected by uranium legacy sites. The territories of potential exposure included Stepnogorsk city, Aksu, Zavodskoy, Kvartsitka, and Bogenbay settlements. Potential exposure pathways include external gamma radiation, inhalation intake of radon and uranium-containing dust particles, as well as possible intake of natural radionuclides and associated chemical pollutants through environmental media.

Individual radiation doses, radon concentrations in residential premises, and uranium or heavy metal content in biological samples of the enrolled patients were not determined within the framework of this study. Therefore, exposure status was established based on long-term residence in the zone of potential influence of tailings storage facilities.

All patients underwent lower extremity revascularization according to current clinical guidelines. The choice of procedure (endovascular, open surgical, or hybrid) was determined by the anatomical characteristics of the lesions and the patient’s overall clinical status. In the study group, 24 patients (68.6%) underwent endovascular interventions (angioplasty), 8 (22.9%) underwent open surgical revascularization (femoro-popliteal bypass), and 3 (8.6%) underwent hybrid procedures. In the control group, 26 patients (74.3%) underwent endovascular interventions, 7 (20.0%) underwent open surgery, and 2 (5.7%) underwent hybrid procedures. No significant differences in the distribution of procedure types were observed between groups (*p* > 0.05).

This study compared the distribution of CLLI stages according to the Fontaine classification before surgery and at 1 month after surgical intervention between the study group and the control group.

### 2.2. Statistical Analysis

Statistical data processing was performed using SPSS Statistics 27 software (IBM Corp., Armonk, NY, USA). To compare the distribution of categorical variables (CLLI stages according to the Fontaine classification) between groups, Pearson’s chi-squared (χ^2^) test was used. In cases where more than 20% of cells in contingency tables had an expected value of less than 5, the likelihood ratio test was additionally calculated. The critical significance level (*p*) was set at 0.05.

## 3. Results

To assess baseline comparability of the groups, an analysis of the distribution of CLLI stages was performed between the study group (*n* = 35) and the control group (*n* = 35) (Table 1). Statistical analysis was performed using Pearson’s chi-squared test in SPSS Statistics 27.

As presented in Table 1, in the study group before surgery, patients with stage III CLLI predominated—18 (51.4%), whereas in the control group, the largest subgroup was those with stage IIb—17 (48.6%). The proportion of patients with critical ischemia (stage IV) was 7 (20.0%) in the study group and 3 (8.6%) in the control group. Despite apparent differences in distribution, Pearson’s chi-squared test did not reveal statistically significant intergroup differences: χ^2^ = 3.688, df = 2, *p* = 0.158. The minimum expected value in the contingency table cells was 5.0, meeting the applicability criteria for the test.

**Figure 1:**
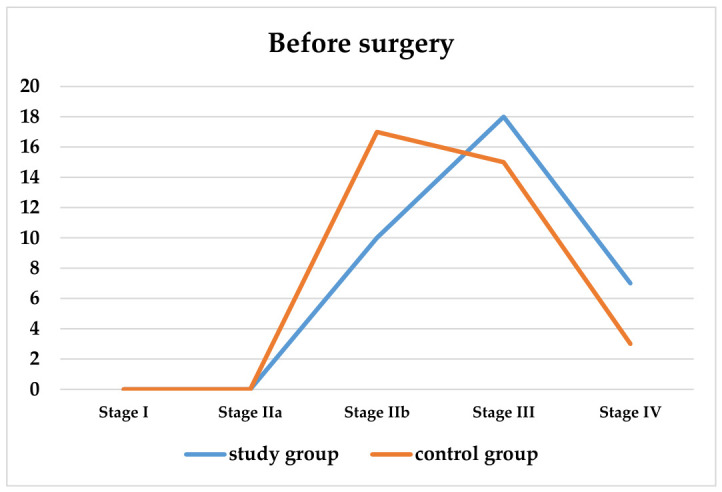
Distribution of patients by preoperative Fontaine stage in the study and control groups.

Thus, no statistically significant differences in baseline Fontaine stage distribution were observed between the study and control groups. However, given the retrospective design and the apparent numerical differences in preoperative disease severity, postoperative findings should be interpreted with caution (Figure 1).

At 1 month after surgical intervention, a comparative analysis of CLLI stage distribution was performed between the study group (*n* = 35) and the control group (*n* = 35). The results are presented in Table 2.

As shown in Table 2, the study group was dominated by patients with stage III—15 (42.8%) and stage IV—7 (20.0%). Only 3 (8.6%) patients in this group showed regression to stage IIa, and no cases of complete regression to stage I were recorded.

In the control group, more favorable dynamics were observed: 16 (45.7%) patients were diagnosed with stage IIa, and 2 (5.7%) with stage I. Severe stages (III–IV) persisted in only 8 (22.9%) patients.

Statistical analysis revealed significant intergroup differences (χ^2^ = 17.547, df = 4, *p* = 0.002). Given that 20% of the contingency table cells had an expected value of less than 5, the likelihood ratio test was additionally calculated, which also confirmed the presence of significant differences (χ^2^ = 19.469, df = 4, *p* = 0.001).

**Figure 2:**
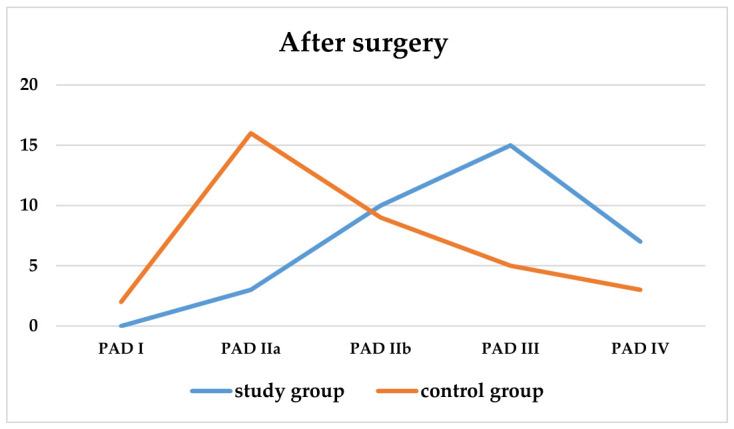
Distribution of patients by CLLI stage after surgery in the study and control groups.

Thus, the control group demonstrated significantly better results in terms of regression of CLLI stages at 1 month after surgery compared to the study group (Figure 2).

## 4. Discussion

This study conducted a comparative analysis of the distribution of chronic lower limb ischemia (CLLI) stages according to the Fontaine classification in patients with LEAD residing in uranium legacy-affected areas (study group, *n* = 35) and patients living outside this zone (control group, *n* = 35). Assessment was performed before surgical intervention and at 1 month postoperatively.

At 1 month after surgery, statistically significant intergroup differences were identified (χ^2^ = 17.547, df = 4, *p* = 0.002). The control group demonstrated significantly greater regression of the disease: the proportion of patients with mild stages (I-IIa) was 51.4% compared to 8.6% in the study group. In contrast, severe stages (III-IV) predominated in the study group—62.8% versus 22.9% in the control group.

Of particular note is the fact that complete regression to stage I was recorded only in the control group (2 patients, 5.7%), whereas no such cases were observed in the study group. This may indicate a less favorable postoperative course of the disease in patients living long-term near the tailings storage facility. From a radiation–hygiene perspective, the less favorable postoperative dynamics in patients residing near uranium tailings storage facilities may be associated with long-term combined exposure to uranium legacy factors and other technogenic pollutants. Chronic low-intensity exposure to such factors may potentially enhance endothelial damage, oxidative stress, inflammatory activation, and microcirculatory disturbances. These mechanisms may contribute to a slower regression of ischemic manifestations and less pronounced recovery after revascularization interventions. It is known from the literature that long-term exposure to heavy metals and other technogenic pollutants may contribute to the accelerated progression of atherosclerosis as well as impaired reparative processes after vascular interventions [5,16]. In patients from the study group who resided for at least 5 years in the zone of potential environmental disadvantage, a more aggressive course of the atherosclerotic process and poorer regenerative capacity of the vascular wall may theoretically be observed.

To date, only a limited number of studies have addressed vascular pathology in populations residing in uranium legacy-affected areas. Most available studies have focused on environmental contamination, radiation–hygiene assessment, general somatic morbidity, and cardiovascular disease prevalence [17,18]. A previous study from Kyrgyzstan analyzed clinical and epidemiological characteristics of obliterating atherosclerosis of lower limb arteries in patients living near uranium legacy sites and reported more frequent involvement of distal arterial segments and differences in reconstructive procedures among exposed patients [14]. However, that study did not specifically evaluate early postoperative regression of ischemia stages after lower extremity revascularization. Therefore, to the best of our knowledge, the present study is among the first to specifically evaluate early postoperative outcomes after revascularization in patients with lower extremity artery disease residing in uranium legacy-affected areas.

Several limitations of the present study should be acknowledged. First, due to the retrospective observational design, detailed information on several important baseline clinical and procedural characteristics was not consistently available for all patients. These included smoking history, dyslipidemia, renal function, lesion complexity, procedural success, and perioperative management. These variables may act as potential confounders and may partly influence postoperative recovery. Therefore, the observed differences in early postoperative ischemia regression cannot be interpreted as independently associated with environmental exposure alone.

Second, exposure status was determined based on long-term residence in the predefined uranium legacy-affected area. However, individual radiation doses, indoor radon levels, heavy metal concentrations, environmental monitoring data, and biological markers of exposure were not assessed in the enrolled patients. Therefore, potential exposure misclassification cannot be excluded. The observed differences in early postoperative regression of CLLI stages should therefore be interpreted as an association rather than evidence of a direct causal relationship. Due to the observational design of the study, the absence of individual exposure assessment, and the lack of adjustment for several demographic and clinical covariates, causality cannot be established. Because of the relatively small sample size and incomplete availability of key clinical covariates, multivariable logistic regression analysis was not performed.

Third, the present study evaluated only early postoperative dynamics of CLLI stages according to the Fontaine classification at 1 month after revascularization. Other clinically important outcomes, including limb salvage, wound healing, repeated revascularization, major adverse limb events, amputation rate, and mortality, were not systematically collected in the available retrospective dataset and therefore could not be analyzed. In addition, the follow-up period was limited to the first postoperative month. Longer follow-up would allow a more complete evaluation of the durability of ischemia regression, limb-related events, repeated interventions, amputation, mortality, and potential long-term vascular consequences associated with residence in uranium legacy-affected areas.

Future prospective studies with larger cohorts, individual exposure assessment, detailed baseline clinical and procedural characterization, adjusted statistical models, and extended follow-up are needed to clarify whether the observed early postoperative differences persist over time and to determine independent predictors of poor postoperative recovery in patients with LEAD residing in uranium legacy-affected areas.

## 5. Conclusions

Patients with LEAD residing long-term in uranium legacy-affected areas demonstrated less favorable early postoperative dynamics compared to patients living outside the zone of potential technogenic exposure. The control group showed more pronounced regression of CLLI stages at 1 month after surgical intervention. However, the identified association requires cautious interpretation, as individual exposure levels and a number of cardiovascular risk factors were not assessed in this study. Further prospective studies with longer follow-up, individual assessment of radiation–hygiene factors and consideration of clinical predictors of postoperative outcomes are needed.

## Figures and Tables

**Table 1:** Comparison of CLLI stage distribution between the study and control groups before surgical intervention.

CLLI Stage	Study Group *n* (%)	Control Group *n* (%)	Statistical Criterion
Stage I	0	0	χ^2^ = 3.688 df = 2 *p* = 0.158
Stage IIa	0	0	
Stage IIb	10 (28.6)	17 (48.6)	
Stage III	18 (51.4)	15 (42.8)	
Stage IV	7 (20)	3 (8.6)	
Total	35 (100)	35 (100)	

Note: Differences between groups are not statistically significant (*p* > 0.05). CLLI, chronic lower limb ischemia. χ^2^—chi-squared test statistic; df—degrees of freedom.

**Table 2:** Comparison of postoperative Fontaine stage distribution between the study and control groups.

CLLI Stage	Study Group *n* (%)	Control Group *n* (%)	Statistical Criterion
Stage I	0	2 (5.7)	χ^2^ = 17.547
Stage IIa	3 (8.6)	16 (45.7)	df = 4
Stage IIb	10 (28.6)	9 (25.7)	*p* = 0.002
Stage III	15 (42.8)	5 (14.3)	
Stage IV	7 (20)	3 (8.6)	
Total	35 (100)	35 (100)	

Note: Differences between groups are statistically significant (*p* < 0.05). CLLI, chronic lower limb ischemia. χ^2^—chi-squared test statistic; df—degrees of freedom.

## Data Availability

The data presented in this study are available on reasonable request from the corresponding author. The data are not publicly available due to privacy and ethical restrictions, as they contain de-identified patient-level clinical data and are subject to institutional approval.

## Abbreviations

The following abbreviations are used in this manuscript:

CLLI	Chronic lower limb ischemia
CVD	Cardiovascular diseases
LEAD	Lower extremity artery disease
UMC CF	Corporate Fund “University Medical Center”
